# Opportunities in cancer gene therapy: inhibiting MDM2 and restoring p14ARF as a means to activate p53

**DOI:** 10.1007/s11010-026-05742-4

**Published:** 2026-09-21

**Authors:** Soraia Barbosa de Oliveira, Ana Laura Vieira Alves, Bianca Naomi Niitsuma, Fernanda Antunes, Gabriel Borel Marinho, Giovanna Lagonegro Amanatidis, Jean Luz, Mariana Barbosa, Pedro Egydio Oliveira Zambotti, Otavio Augusto Rodrigues, Bryan E. Strauss

**Affiliations:** 1 Faculdade de Medicina, Centro de Investigação Translacional em Oncologia (CTO)/LIM24, Comprehensive Center for Precision Oncology (C2PO), Instituto do Câncer do Estado de São Paulo (ICESP), Universidade de São Paulo (FMUSP), Universidade de São Paulo, São Paulo, Brazil; 2 Laboratório de Vetores Virais (LVV), Faculdade de Medicina, Centro de Investigação Translacional em Oncologia (CTO), Instituto do Câncer do Estado de São Paulo (ICESP), Universidade de São Paulo (FMUSP), Av. Dr. Arnaldo, 251, 8o Andar, Cerqueira César, São Paulo, SP 01246-000 Brazil

**Keywords:** Gene therapy, Tumorigenesis, p53, MDM2, P14ARF

## Abstract

The MDM2/p53/p14ARF axis represents a central regulatory network controlling cell-cycle progression, genomic stability, and tumor suppression, and is frequently deregulated in human cancers. In many malignancies, particularly those retaining wild-type *TP53*, p53 activity is functionally suppressed through MDM2 overexpression or CDKN2A (p14ARF) loss, events that may be reversed in order to activate this pathway. This review explores the biological basis of the MDM2/p53/p14ARF axis and its relevance as a target for gene therapy and multimodal treatment strategies. While gene therapy for the restoration of p53 has been extensively explored, the use of genetic interventions for the inhibition of MDM2 or the restoration of p14ARF is underrepresented, though they have the potential to re-establish DNA damage responses, promote apoptosis, and enhance tumor sensitivity to chemotherapy and radiotherapy. In addition, p53 activation is now recognized as a contributor to immunogenic cell death and modulation of the tumor microenvironment. Laboratory and early clinical studies demonstrate that inhibition of MDM2 and restoration of p14ARF by gene transfer can integrate tumor cytotoxicity with immune activation. Although large-scale clinical validation remains limited, current evidence supports the MDM2/p53/p14ARF axis as a promising, context-dependent target within biomarker-driven combination therapies rather than a standalone approach.

## Introduction

Cancer is classically defined as the uncontrolled and unregulated proliferation of cells with the capacity to disseminate throughout the body [1]. Tumor cells can modify their phenotypic state in response to environmental conditions, proliferate without regulatory control, compromise normal tissue function, and induce inflammatory processes, contributing to resistance to current therapeutic approaches [2]. Although somatic mutations may arise in normal tissues, tumor suppressor genes normally eliminate altered cells; cancer develops when failures in these regulatory mechanisms allow genetically and epigenetically altered cells to accumulate and proliferate [3].

Conventional cancer therapies, including cytotoxic chemotherapy and radiotherapy, present limited efficacy due to low tumor specificity, systemic toxicity, and rapid selection of resistant clones, processes strongly associated with intratumor heterogeneity and genomic instability of malignant neoplasms [4, 5]. Targeted therapies were developed to interfere with oncogenes or signaling pathways involved in tumor initiation and maintenance; however, their clinical benefit is frequently transient because of pathway redundancy, compensatory signaling activation, and the emergence of resistant or drug-tolerant subclones [6–8]. The genomic complexity of tumors therefore limits single-target approaches, as inhibition of isolated components of oncogenic networks is often insufficient to produce durable therapeutic responses [5, 9].

Within this framework, therapeutic strategies aimed at restoring disrupted tumor suppressor networks, including gene therapy approaches, have emerged as an alternative to isolated inhibitory treatments [10]. Gene therapy is defined as the therapeutic transfer or modulation of genetic material into target cells to alter the course of a disease [11]. One established strategy involves recombinant adenoviral vectors encoding wild-type *TP53* to restore p53 activity, with clinical studies demonstrating therapeutic efficacy in several cancers, including head and neck squamous cell carcinoma, nasopharyngeal carcinoma, and cervical cancer [12]. Since p53 acts on many cellular targets, its restoration is expected to activate a variety of anti-cancer activities, thus providing a better chance of controlling tumor progression as compared to interventions that target a single factor. However, the complex regulatory network that influences p53 activity, particularly MDM2 (mouse double minute 2), must be taken into consideration. Inhibition of MDM2 is an attractive target for cancer intervention, including gene therapy, since many cancers retain wild-type p53 (WTp53) that is non-functional due to elevated levels of MDM2. Loss of p14ARF (alternative reading frame of CDKN2A), a critical inhibitor of MDM2 activity, also occurs in many cancers, implying that its restoration is also expected to activate the p53 pathway. Here we detail the MDM2/p53/p14ARF axis and underrepresented opportunities for cancer gene therapy involving the inhibition of MDM2 and/or restoration of p14ARF.

## Tumor suppressor functions of p53

Loss of tumor suppressor function contributes to tumorigenesis by disrupting cell-cycle checkpoints, DNA repair mechanisms, and apoptotic pathways. Uncontrolled proliferation compromises DNA replication fidelity, generating replication stress and accumulation of DNA damage [13, 14]. Replication stress is recognized as an early event in malignant transformation and a major source of genomic instability [15]. Preservation of genomic stability is essential for cellular homeostasis and tissue integrity [5], and the MDM2/p53/p14ARF signaling axis represents one of the best-characterized stress-response circuits linking DNA damage sensing to cell-cycle regulation and DNA repair. A central function of p53 is to maintain replication fidelity and prevent transmission of damaged DNA during mitosis [16].

*TP53* encodes p53, a transcription factor and the most frequently altered tumor suppressor in human cancers [17, 18]. It is activated by multiple forms of cellular stress, including DNA double-strand breaks, replication stress, oncogene activation, and oxidative damage [19]. Once activated, p53 orchestrates transcriptional programs that promote temporary cell-cycle arrest, facilitate DNA repair, or induce apoptosis or senescence when damage cannot be resolved, thereby restricting the propagation of genetically unstable cells and preserving genomic integrity [20]. In parallel, p53 influences additional processes such as metabolism, angiogenesis, and immune regulation, all of which contribute to its tumor-suppressive function [20, 21].

P53 protein levels and activity are controlled through multiple post-translational mechanisms, including ubiquitin-dependent mechanisms, among which MDM2 is one of the best-characterized E3 ubiquitin ligases. MDM2 promotes p53 ubiquitination and targets it for proteasomal degradation [22]. Because p53 transcriptionally activates MDM2, this establishes a negative feedback loop that maintains low p53 activity in unstressed cells. Importantly, MDM2-mediated regulation of p53 under low-stress conditions is not limited to proteasomal degradation, as MDM2 can also promote NEDD8 conjugation to p53, thereby inhibiting its transcriptional activity and stabilizing an inactive form of the protein [23]. In response to DNA damage, post-translational modifications such as phosphorylation and acetylation interfere with the p53–MDM2 interaction, leading to p53 stabilization and activation of its transcriptional functions [24]. Beyond stabilization, specific phosphorylation events further dictate p53 functional outcomes. In particular, phosphorylation at serine 46 and threonine 81 has been associated with the selective activation of pro-apoptotic target genes [25]. Oncogenic stress signals, primarily mediated through p14ARF, not only stabilize p53 by inhibiting MDM2 but also contribute to the modulation of these stress-specific post-translational modifications, thereby promoting apoptotic responses. Together, these interactions form a regulatory network that fine-tunes both the intensity and qualitative output of p53 signaling to maintain cellular homeostasis [25–27].

A critical component of p53 function is its role in checkpoint control. Loss of p53 disrupts the G1 checkpoint, allowing cells with damaged DNA to enter S phase [28], and is strongly associated with genomic instability [15]. Genomic analyses indicate that approximately 40–50% of tumors harbor TP53 mutations, most commonly missense changes within the DNA-binding domain that impair its transcriptional activity. Some of these mutations also confer gain-of-function properties that promote tumor progression [29]. Such alterations perturb the MDM2/p53/p14ARF regulatory axis. While mutant p53 can still be targeted by MDM2, certain variants exhibit increased stability and accumulation. Loss-of-function mutations compromise genome surveillance, whereas gain-of-function variants can reprogram transcriptional and chromatin landscapes, altering pathways involved in cellular homeostasis [30]. In addition, some mutant forms exert dominant-negative effects over the wild-type protein [21].

Beyond its classical roles, p53 also contributes to immune-related processes. It supports immunogenic cell death by promoting the release of danger-associated molecular patterns, including ATP and HMGB1, and by facilitating calreticulin exposure on the cell surface, which enhances the activation of antigen-presenting cells and the initiation of adaptive immune responses [31, 32]. p53 further influences antigen presentation by regulating genes involved in MHC class I pathways and antigen-processing machinery, thereby improving recognition of tumor cells by cytotoxic T lymphocytes [33]. It can also modulate the expression of ligands recognized by natural killer cells, reinforcing immune surveillance mechanisms.

**Fig. 1:**
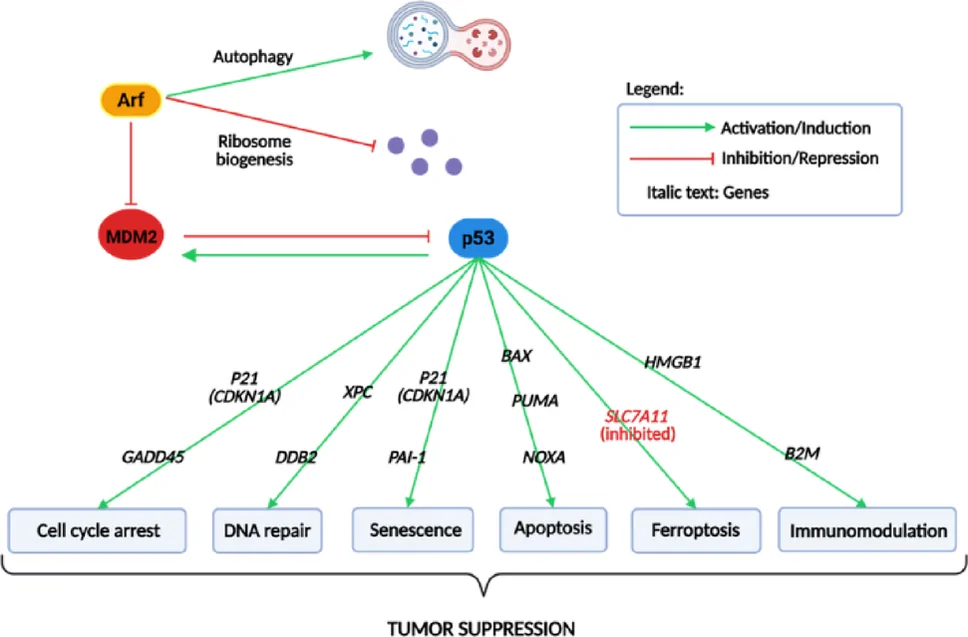
Integrated signaling network of p14ARF and p53-mediated tumor suppression. The central MDM2/p53/p14ARF regulatory triangle governs the cellular response: p14ARF inhibits MDM2, preventing p53 degradation, while p53 completes the feedback loop by transactivating MDM2. Beyond this core, p14ARF independently regulates autophagy and ribosome biogenesis. Upon stabilization, p53 transactivates genes for cell cycle arrest (p21, GADD45), DNA repair (XPC, DDB2), senescence (p21, PAI-1), and apoptosis (BAX, PUMA, NOXA). Additionally, p53 triggers ferroptosis by inhibiting SLC7A11 and immunomodulation, through increased expression of HMGB1 and B2M, stabilizes the MHC class I complex, among others, collectively ensuring potent tumor suppression

At the tissue level, p53 shapes the tumor microenvironment through regulation of cytokines and chemokines, affecting immune cell recruitment and inflammatory signaling. In addition to its immunomodulatory functions, p53 also contributes to the control of tumor angiogenesis by inducing anti-angiogenic programs and regulating genes involved in vascular remodeling, including endogenous angiogenesis inhibitors such as thrombospondin-1, BAI1, endostatin, and tumstatin [34]. Loss or functional attenuation of p53 may therefore favor a pro-angiogenic tumor microenvironment, not only by impairing immune surveillance but also by reducing anti-angiogenic gene expression. Evidence also suggests that p53-dependent induction of anti-angiogenic factors may require cooperation with other tumor suppressors, such as VHL. In this context, MDM2-mediated neddylation of VHL has been reported to impair the p53–VHL interaction and block the induction of anti-angiogenic factors, highlighting an additional mechanism by which MDM2 may promote angiogenesis independently of canonical p53 degradation [35]. Functional p53 is generally associated with conditions that favor immune activity, whereas its loss can contribute to immune evasion through altered signaling networks [36, 37]. Moreover, p53 has been linked to the regulation of immune checkpoint pathways, including PD-L1 expression and T cell function, connecting cellular stress responses with immune control [38]. As shown in Fig. 1 these p53 features are central coordinators of cellular stress responses, integrating genome maintenance, metabolic regulation, and immune surveillance within its core biological roles.

### MDM2 and p14ARF: key regulators of p53

Given the central role of p53 in determining cell fate under oncogenic stress, regulatory components of the MDM2/p53/p14ARF axis are essential for preserving anti-tumorigenic mechanisms. MDM2 and p14ARF form a coordinated regulatory pair that modulates p53 activity according to cellular demands, ensuring appropriate activation or inhibition of stress responses [39].

**Fig. 2:**
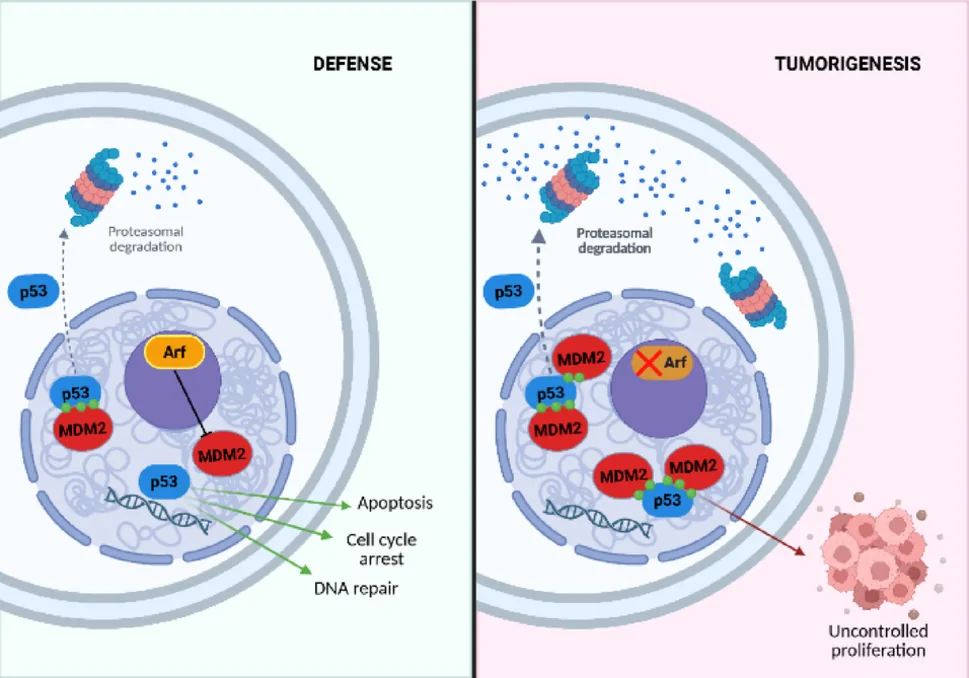
Dysregulation of the MDM2/p53/p14ARF axis in cancer. Comparative schematic of p53 regulation. As a defense upon oncogenic assault (left), p14ARF sequesters MDM2 in the nucleolus, stabilizing p53 and maintaining cellular control. In tumorigenesis (right), the loss of p14ARF leads to an excess of free MDM2, which binds to p53 and targets it for proteasomal degradation. The resulting depletion of p53 levels impairs tumor suppression, leading to uncontrolled cell proliferation

MDM2, also referred to in humans as HDM2, is encoded on chromosome 12q14.3–q15 and consists of 491 amino acids organized into functional domains, including an N-terminal p53-binding domain, an acidic region, a zinc-finger motif, a RING finger domain, and nuclear localization and export signals [40, 41]. As a member of the E3 ubiquitin ligase superfamily, MDM2 functions as an adaptor that links substrate proteins to E2 ubiquitin-conjugating enzymes, thereby regulating protein localization, activity, and degradation [42, 43]. Notably, MDM2 E3 ligase activity is dynamically regulated by post-translational modifications, particularly phosphorylation, which regulates its interaction with distinct E2 enzymes. Under basal conditions, MDM2 preferentially associates with ubiquitin-conjugating enzymes such as UBC5 to promote substrate ubiquitination and proteasomal degradation. However, under growth-promoting conditions, phosphorylation of MDM2 by c-Src at tyrosine residues Y281 and Y302 induces a functional switch in E2 usage, favoring interaction with UBC12, the NEDD8-conjugating enzyme. This switch converts MDM2 from a ubiquitin E3 ligase into a NEDD8 E3 ligase, enabling the neddylation of substrates including p53. Consequently, neddylated p53 exhibits increased stability but reduced transcriptional activity, thereby contributing to the accumulation of an inactive p53 pool. This phosphorylation-dependent modulation of E2 selection highlights an additional regulatory layer that expands the functional repertoire of MDM2 beyond canonical ubiquitin-mediated proteolysis [44, 45] (Fig. 2).

Within the p53 regulatory network, MDM4 (also known as MDMX) functions as a critical co-regulator of MDM2. Although MDM4 lacks intrinsic E3 ubiquitin ligase activity, it binds directly to p53 and inhibits its transcriptional activity. MDM4 also heterodimerizes with MDM2, facilitating the interaction of the complex with E2 ubiquitin-conjugating enzymes and promoting p53 ubiquitination. Monoubiquitination of p53 is associated with its nuclear export, whereas subsequent polyubiquitination events can target p53 for proteasomal degradation. Thus, rather than directly mediating p53 degradation, the MDM2–MDM4 complex regulates p53 stability and subcellular localization through coordinated ubiquitination processes. Elevated MDM4 expression has been associated with reduced sensitivity to MDM2-targeted therapies and may contribute to therapeutic resistance [42, 43].

Since its identification in 1987, MDM2 has been recognized as a key regulator of cell-cycle progression, apoptosis, DNA repair, and oncogenic signaling, primarily through its interaction with p53. Under stress conditions such as DNA damage, hypoxia, or oncogene activation, WTp53 induces transcriptional programs that promote tumor suppression and elimination of damaged cells. MDM2 restrains p53 through multiple mechanisms, including direct inhibition of its transactivation domain, regulation of nuclear export, and ubiquitin-mediated proteasomal degradation [40, 41, 46]. Because MDM2 is transcriptionally induced by p53, these activities establish a negative feedback loop that maintains low basal p53 levels and prevents inappropriate cell-cycle arrest or apoptosis [39, 47]. The relationship is not exclusively inhibitory, however: MDM2 can also bind p53 mRNA via its C-terminal RING domain and enhance its translation, particularly under stress conditions, adding a positive arm to this circuit [48].

Balanced regulation of p53 and MDM2 is essential for maintaining cellular homeostasis. In addition to its canonical role in controlling p53 stability and activity, MDM2 overexpression has been implicated in multiple tumor-promoting processes through the modulation of signaling pathways that extend beyond p53 regulation. MDM2 interacts with components of the retinoblastoma (Rb) and transforming growth factor-β (TGF-β) pathways, contributing to cell-cycle deregulation, epithelial–mesenchymal transition (EMT), and the activation of pro-metastatic signaling programs. Furthermore, MDM2 has been associated with metastatic dissemination through the regulation of cytoskeletal dynamics, cell motility, and invasive behavior. Emerging evidence also supports a role for MDM2 in angiogenesis through modulation of hypoxia-inducible factor-1α (HIF-1α) signaling and the expression of pro-angiogenic mediators such as vascular endothelial growth factor (VEGF). Beyond these functions, MDM2 has been implicated in metabolic reprogramming, evasion of apoptosis, and modulation of anti-tumor immune responses. In addition, MDM2 contributes to the regulation of cellular senescence and aging through its effects on genomic stability and DNA damage response pathways. Dysregulation of these processes may promote genomic instability, tumor progression, and increased tumor aggressiveness [40, 49–51].

Amplification or overexpression of MDM2 has been reported across a wide spectrum of malignancies, including sarcomas, melanomas, leukemias, lymphomas, glioblastomas, and carcinomas of the lung, breast, liver, esophagogastric region, and colon. In these contexts, increased MDM2 activity contributes to functional inactivation of p53, even in the absence of *TP53* mutations [16, 52, 53].

The ARF protein is encoded by the CDKN2A locus on chromosome 9p21 and is produced from an alternative first exon under its own promoter, independent of p16INK4a, generating a 132–amino acid arginine-rich protein. In humans, this isoform is referred to as p14ARF, whereas in mice it is referred to as p19Arf. Although p14ARF and p19Arf are functional orthologs and share conserved tumor suppressor roles, species-specific differences and context-dependent mechanistic variations have been reported, particularly in experimental murine models, highlighting caution in direct extrapolation of mouse ARF biology to human p53 regulatory networks. In both systems, ARF primarily regulates p53 activity through inhibition of upstream negative regulators, particularly MDM2, although additional context-dependent interactions have been described in murine models. Despite these differences, ARF proteins function as key sensors of oncogenic and cellular stress signals, including MYC, RAS, E2F1 activation, heat shock, and oxidative stress. Through these pathways, ARF contributes to cell-cycle regulation across G1, S, and G2 phases via both p53-dependent and p53-independent mechanisms [54, 55].

Within the p53 signaling pathway, E3 ubiquitin ligases play a central role in regulating p53 stability and activity through ubiquitination-mediated degradation. Several E3 ligases contribute to the negative regulation of p53, including MDM2 and PIRH2, which are direct transcriptional targets of p53 and function in autoregulatory negative-feedback loops, as well as COP1 and Mule/ARF-BP1 (HUWE1), which are not generally considered direct p53-responsive genes. Although these proteins are essential for maintaining normal cellular homeostasis, their overexpression or dysregulation can contribute to tumorigenesis by attenuating p53-dependent tumor suppressor functions [19, 56, 57].

Upon oncogenic activation, p14ARF functions as a fail-safe mechanism that counteracts excessive p53 inhibition. In response to aberrant activation of oncogenes such as MYC, RAS, or E2F1, p14ARF expression is induced, leading to the inhibition of key negative regulators of p53, particularly MDM2 and ARF-BP1/Mule. ARF-mediated sequestration and inhibition of MDM2 prevent p53 ubiquitination and proteasomal degradation, resulting in p53 stabilization and activation of transcriptional programs involved in cell-cycle arrest, senescence, and apoptosis [19, 56].

ARF also contributes to tumor suppression through its functional interaction with the Rb/E2F pathway. In response to oncogenic or hyperproliferative signals, E2F1 becomes activated and induces the expression of several tumor suppressor factors, including ARF and TAp73. E2F1 is a direct transcriptional activator of TP73, leading to increased TAp73 expression and activation of apoptotic programs [58, 59]. Simultaneously, ARF promotes p53-dependent tumor suppressive responses by binding to MDM2 and limiting its inhibitory effects on p53 [39]. Through these coordinated mechanisms, oncogenic signaling can activate both the ARF–MDM2–p53 and E2F1–TAp73 pathways. Because TAp73 and p53 share numerous transcriptional targets involved in apoptosis, DNA damage responses, and cell-cycle regulation, these pathways cooperate to promote tumor suppression. Consequently, activation of E2F1 can engage both p53-dependent and p53-independent tumor suppressor programs, providing multiple barriers against malignant transformation [59].

In addition, p14ARF directly interacts with and inhibits the HECT-domain E3 ubiquitin ligase ARF-BP1/Mule. Under basal conditions, Mule contributes to p53 ubiquitination and turnover. Consequently, ARF-mediated inhibition of Mule further stabilizes p53, prolonging its half-life and enhancing the transcription of downstream target genes, including CDKN1A (p21WAF1/CIP1), PMAIP1 (NOXA), and BAX [56, 60]. Through the coordinated inhibition of both MDM2 and Mule, p14ARF amplifies p53-dependent tumor suppressor responses following oncogenic stress. Beyond its regulation of p53, Mule also targets several additional substrates, including Mcl-1, c-Myc, and proteins involved in DNA repair. Therefore, inhibition of Mule by p14ARF may contribute to both p53-dependent and p53-independent tumor suppressor mechanisms [61, 62].

Disruption of this regulatory equilibrium is frequent in human cancers. Deletions, point mutations, or epigenetic silencing of CDKN2A are observed in multiple solid tumors, including glioblastoma, pancreatic carcinoma, adenocarcinoma, and melanoma [63, 64]. P14ARF suppression can also occur independently of genomic alterations through post-translational mechanisms, weakening inhibition of MDM2 and enabling persistent p53 degradation even in tumors retaining wild-type *TP53* [54].

Functional deregulation of p14ARF may also result from altered protein interactions. Nucleophosmin (NPM1), a nucleolar chaperone, stabilizes and retains p14ARF within the nucleolus under physiological conditions. Mutations in NPM1 can induce cytoplasmic relocalization, sequestering p14ARF away from MDM2 and impairing its tumor suppressor activity without CDKN2A genomic alteration [64]. Additionally, proteasomal degradation of p14ARF via CRL2-KLHDC3-dependent ubiquitination has been described in non–small cell lung carcinoma. Loss of p14ARF relieves its inhibitory effects on MDM2, leading to enhanced suppression of p53 signaling and attenuation of downstream p53-dependent tumor suppressive responses despite an intact CDKN2A locus [65].

Collectively, these findings underscore the importance of coordinated regulation within the MDM2/p53/p14ARF axis for maintaining tumor suppressor function and illustrate multiple mechanisms by which its disruption contributes to malignant progression.

## The MDM2/p53/p14ARF axis as a target for gene therapy

Within the field of cancer gene therapy, one of the most extensively investigated molecular targets has been p53. A major milestone was the regulatory approval in China, in 2003, of Gendicine, a recombinant adenoviral vector-based p53 gene therapy authorized for the treatment of head and neck squamous cell carcinoma. This approval marked the first commercial gene therapy product worldwide and provided clinical proof-of-concept that restoration of p53 function could yield therapeutic benefit [12].

Although p53-directed strategies have been widely investigated, contemporary approaches involve combined and integrative therapeutic platforms that have renewed interest in modulation of the MDM2/p53/p14ARF axis for cancer therapy. Given the central role of this pathway in cell cycle regulation, DNA damage responses, apoptosis, and tumor-immune interactions, simultaneous or context-dependent targeting of p14ARF, MDM2, and p53 represents a rational and promising strategy for next-generation gene therapy development, particularly within combination-based and precision oncology frameworks. While we mention p53 gene therapy here, our goal is to highlight the as yet untapped potential of inhibiting MDM2 or restoring p14ARF using genetic interventions as a means to promote anti-tumor responses through p53 dependent and independent mechanisms.

It is important to distinguish gene-based approaches from the broader landscape of p53-targeted therapies. Pharmacological strategies, particularly small-molecule and peptide-based agents, constitute the most extensively developed approaches for modulating the p53 pathway. These compounds have primarily been designed to disrupt the MDM2–p53 interaction, stabilize wild-type p53, or reactivate mutant p53. In contrast, gene transfer represents a smaller, although clinically and biologically relevant, subset of p53-directed therapeutic approaches. The present review therefore focuses specifically on genetic strategies that restore or modulate components of the MDM2/p53/p14ARF axis, with particular emphasis on p53 gene transfer, p14ARF restoration, and genetic inhibition of MDM2. This distinction provides an important context for the scope of this review: while pharmacological modulation of the p53 pathway has been extensively investigated, the comparatively smaller body of gene therapy research highlights opportunities that remain less explored, particularly the genetic inhibition of MDM2 and restoration of p14ARF [66–68].

### Gene therapy targeting p53

Gene therapy approaches targeting p53 generally aim to restore WTp53 activity in tumor cells, overcome mutant p53 (MTp53) or activate downstream p53 signaling pathways. Over the past decades, several delivery platforms and therapeutic strategies have been developed to exploit the tumor suppressive functions of p53 for cancer treatment [69]. The most extensively studied approach is WTp53 gene therapy, which involves delivering a functional *TP53* gene into tumor cells to restore its biological activity. This strategy is most commonly implemented using viral vectors, particularly recombinant adenoviruses due to their high transduction efficiency and ability to deliver large genetic payloads [69, 70]. Although retroviral vectors were used in early studies to deliver p53 [71], results have shown that retrovirus was not an appropriate vector for in vivo/in situ applications and was therefore soon replaced by adenovirus (Ad) [72].

One of the most prominent examples is the recombinant, non-replicating serotype 5 adenoviral vector encoding WTp53 (rAd-p53), Gendicine. Following transduction of tumor cells, the vector proves overexpression of functional p53, triggering apoptosis, growth arrest, or senescence in cancer cells [72]. Preclinical studies in cancer cell lines and animal models demonstrated that intratumor administration of rAd-p53 induced p53 expression, apoptosis, cell-cycle arrest, and tumor growth inhibition. Clinical studies have reported tumor responses, particularly when rAd-p53 was combined with chemotherapy or radiotherapy [67, 72, 73]. After the approval of Gendicine in 2003, its application for other types of tumors such as cervical cancer, non-small cell lung cancer, lung adenocarcinoma, has been evaluated in various clinical studies. Clinical observations from nearly 30,000 people treated indicate 30% to 40% complete response rate and a 50% to 60% partial response rate, particularly when rAd-p53 is used in combination with conventional therapies such as chemotherapy, radiotherapy or other therapeutic regimens [12, 73, 74]. Other adenoviral p53 gene therapies that were evaluated in the clinical setting include INGN 201 (Advexin) for the treatment of esophageal squamous cell carcinoma [75] and SCH-58,500 (ACN53) for recurrent ovarian cancer [76] but despite encouraging findings, the therapeutic efficacy observed was insufficient to achieve regulatory approval [77].

Another promising strategy involves oncolytic viruses engineered to express p53, which combine tumor-selective viral replication with restoration of p53 tumor suppressor activity. These conditionally replicating adenoviruses are vectors designed to preferentially replicate in cancer cells, leading to direct oncolysis while simultaneously delivering therapeutic genes as p53 gene [69, 70]. A representative example is the oncolytic adenovirus OBP-702, a modified version of the telomerase-dependent virus OBP-301 (Telomelysin). In this system, viral replication is driven by the human telomerase reverse transcriptase (hTERT) promoter, which is highly active in tumor cells but largely inactive in normal tissues, thereby conferring tumor specificity [78]. OBP-702 additionally contains a p53 expression cassette inserted into the viral genome, enabling enhanced antitumor activity through the induction of apoptosis, autophagy, and immunogenic cell death (ICD) in tumor cells. This process is associated with the release of danger-associated molecular patterns and increased tumor immunogenicity, contributing to activation of antitumor immune responses. In pancreatic ductal adenocarcinoma (PDAC) preclinical models, OBP-702 has demonstrated antitumor activity in both tumor cell lines and animal models. In cancer cell lines, OBP-702 induces cell death through p53 restoration, viral replication, apoptosis, autophagy, and immunogenic cell death. In animal models, these effects are associated with tumor growth inhibition and modulation of the tumor microenvironment, including effects on pancreatic stellate cells, which contribute to tumor fibrosis, chemoresistance, and immune evasion, and may facilitate viral spread within the tumor microenvironment, thereby enhancing therapeutic efficacy. Notably, these immunogenic effects have been associated with improved responses to immune checkpoint blockade, including PD-1/PD-L1 inhibition, supporting a synergistic strategy that combines viral therapy, p53 restoration, and immunotherapy [78–81].

Beyond viral vectors, non-viral gene delivery platforms have been explored to improve safety and reduce immunogenicity associated with viral gene therapy. These approaches include liposomal nanoparticles, polymeric carriers, dendrimers, and inorganic nanoparticles designed to transport p53-encoding DNA into tumor cells [69]. One example that has been investigated is SGT-53, a transferrin receptor-targeted liposomal nanoparticle designed to selectively deliver *TP53* DNA to tumor cells that overexpress transferrin receptors. Early-phase clinical trials in patients with advanced solid tumors demonstrated that SGT-53 is well tolerated and capable of achieving tumor-targeted gene delivery, supporting its continued clinical development [82]. Numerous other nanocarrier systems, including polymeric nanoparticles, gold nanoparticles, and mesoporous silica nanomaterials, have shown promising preclinical results by enhancing p53 transfection efficiency and inducing apoptosis in tumor cells [69].

In addition to DNA replacement therapy, emerging approaches aim to modulate p53 signaling through RNA-based therapeutics and gene editing technologies [69]. For example, p53 mRNA therapy has been proposed as a method to transiently increase WTp53 protein levels in tumor cells without the risk of genomic integration. Additionally, small interfering RNAs (siRNAs) targeting specific MTp53 variants have been designed to selectively silence mutant alleles, thereby restoring the tumor suppressive activity of endogenous WTp53 [83]. More recently, CRISPR-Cas9 genome editing has been explored as a strategy to correct *TP53* mutations or restore normal gene function, although concerns regarding off-target effects and genomic instability remain significant obstacles to clinical translation [84, 85]. In the field of vaccines, rVV-TK-53 was tested in bladder cancer cells to transfer p53 using vaccine virus vectors and achieved prolonged survival of mice [86], but the studies did not continue to clinical trials. Another example is Ankara (p53MVA) that has been evaluated in clinical trials with promising antitumor effects [87].

Despite promising results, p53 gene therapy often shows limited efficacy as a monotherapy, which has led to increasing interest in combination strategies. Restoration of p53 activity can sensitize tumor cells to chemotherapy, radiotherapy, and immunotherapy by promoting apoptosis and suppressing mechanisms of drug resistance [69, 88]. For example, WTp53 has been shown to downregulate multidrug resistance-associated genes, including MDR1 and members of the ABC transporter family, thereby enhancing the efficacy of cytotoxic agents such as cisplatin, paclitaxel, and gemcitabine [89]. In a study from our group, we have shown that non replicative adenovirus with improvements at the level of transduction and transcription encoding human p53 (AdRGD-PG-p53), in a prostate cancer xenograft model, combination of AdRGD-PG-p53 with a sub-therapeutic dose of cabazitaxel reduced tumor progression and increased survival to 100% [90]. In cancer cell line models, p53 gene transfer increased radiosensitivity, as evidenced by increased p21 expression, caspase activation, and apoptosis; in corresponding in vivo studies, these effects were associated with inhibition of tumor growth [91]. However, one concern about reestablishing p53 in tumor cells is the possibility that DNA damage from chemotherapy or radiotherapy would be detected as a danger signal and, in response, activate DNA repair mechanisms in the tumor cells, leading to treatment resistance [69]. This topic has been reviewed previously [91], but a recent study showed that p53 can activate the circular RNA circASCC3, thus preventing R-loop accumulation, favoring DNA repair, and allowing for cell survival after treatment with cisplatin or etoposide [92].

Furthermore, p53 activation can stimulate anti-tumor immune responses and improve the effectiveness of immune checkpoint inhibitors such as anti-PD-1 antibodies. Indeed, recent studies suggest that p53-expressing viruses or p53 mRNA nanoparticles can induce immunogenic cell death and reduce immunosuppressive cell populations within tumors, thereby potentiating antitumor immune responses [93–95].

Although the p53 pathway represents a promising therapeutic target, several limitations may compromise the efficacy of p53-based gene therapy. One important challenge is that p53 exists in multiple isoforms, including Δ40p53, Δ133p53, and Δ160p53, which can exert dominant-negative or even pro-tumorigenic effects by interfering with canonical wild-type p53 (WTp53) signaling [96]. Consequently, the introduction of exogenous WTp53 may not fully restore tumor suppressor activity. Another major limitation is that many cancers harbor alterations in downstream components of the p53 signaling pathway. Therefore, even when gene therapy successfully delivers functional WTp53, apoptosis may not occur if critical mediators such as p21, BAX, PUMA, NOXA, APAF1, or caspases are deleted, mutated, or epigenetically silenced. Furthermore, hyperactivation of oncogenic pathways, including PI3K/AKT/mTOR, MAPK/ERK, MYC, WNT/β-catenin, and NF-κB, can override p53-mediated growth suppression and sustain tumor cell survival despite p53 restoration. In addition, overexpression of the negative regulators MDM2 and MDM4 may rapidly neutralize the newly introduced WTp53 protein before a sufficient antitumor response can be achieved [36]. Off-target effects are also an important concern, as excessive or ectopic p53 activation in normal tissues may induce apoptosis, senescence, or tissue damage. And, as mentioned above, p53 restoration can, under certain circumstances, promote DNA repair rather than apoptosis following chemotherapy or radiotherapy, thereby enabling some tumor cells to survive genotoxic stress instead of undergoing cell death [36].

These limitations may partially explain why FDA has not yet approved such approaches for oncologic treatment highlighting the need for more studies to develop new p53 gene therapy strategies.

### p14ARF as a potential therapeutic target

The biological characteristics of p14ARF as a central regulator of the MDM2/p53 axis, position it as a particularly compelling candidate for gene therapy-based intervention. As an intrinsically disordered protein lacking catalytic activity and functioning primarily through protein-protein interactions, p14ARF is not readily amenable to small-molecule pharmacological activation [97, 98]. The absence of enzymatic pockets or defined structural domains limits conventional drug discovery strategies and helps explain the lack of clinical research or approved drugs that act directly on p14ARF (Table 1). Gene delivery platforms, by contrast, enable controlled expression of the full-length functional protein, thereby bypassing structural constraints that hinder pharmacological modulation.

In this setting, gene therapy may represent a mechanistically plausible strategy to modulate p14ARF. Laboratory studies suggest that controlled reintroduction or restoration of p14ARF expression can counteract MDM2-mediated inhibition of p53 and reactivate downstream tumor suppressive programs. In this review, we argue that such approaches may, in principle, help overcome functional impairments caused by altered protein stability, subcellular mis-localization, or proteasomal degradation of pathway components. Although still largely experimental, gene-based strategies targeting components of the p53 regulatory network may provide a framework for future combinatorial approaches aimed at overcoming tumor-associated defects in p53 pathway signaling.

**Table 1:**
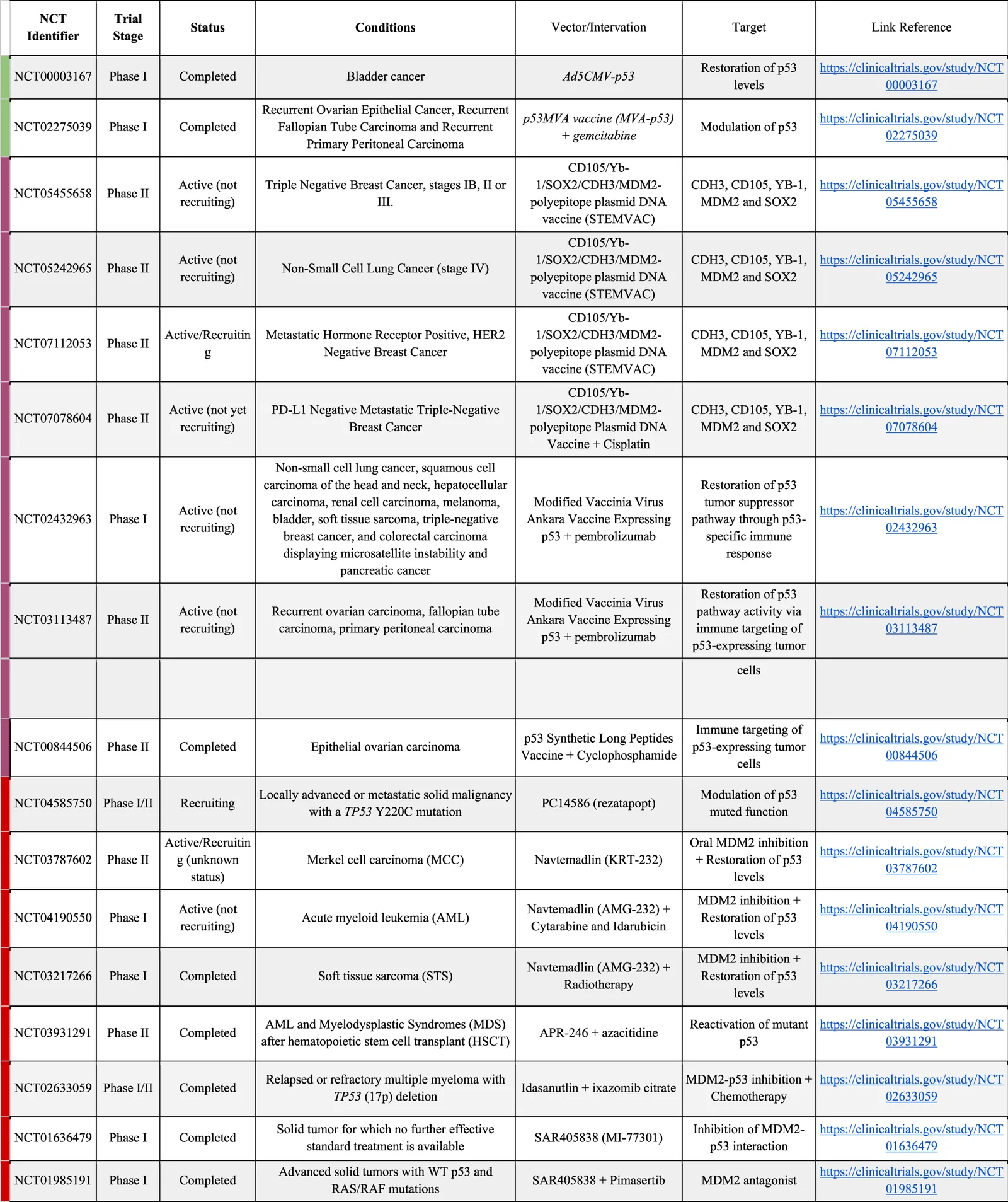
Selected clinical trials prioritizing gene-based therapies involving p53, p14ARF, and MDM2 across different cancer types

For each study, key information is provided, including the NCT identifier, clinical trial phase, current recruitment status, study start date, targeted condition, vector or therapeutic intervention, molecular target, and a reference link for access to the official trial registry. To facilitate interpretation, a color-coded system was applied: green indicates active or successfully completed studies; purple highlights vaccine-based approaches; and red denotes studies involving pharmacological interventions

### Gene therapy for the restoration of p14ARF

Preclinical studies of p14ARF gene therapy have evaluated its activity in both cancer cell lines and animal models, demonstrating that therapeutic efficacy depends strongly on tumor genotype and pathway integrity. In cancer cell-line models retaining functional *TP53*, adenoviral-mediated p14ARF expression promotes cell-cycle arrest and apoptosis through inhibition of MDM2 and restoration of p53 signaling. In vivo, p14ARF gene transfer has been shown to reduce tumor growth and induce cytotoxic effects; however, these responses are generally incomplete and infrequently result in sustained tumor regression [100, 101]. Restoring ARF is also discussed in recent work involving pancreatic ductal adenocarcinoma (PDAC), in which inactivation of CDKN2A/ARF promotes tumor progression and remodeling of the tumor microenvironment. These studies indicate that ARF can exert tumor-suppressive effects beyond its canonical regulation of p53, including p53-independent modulation of the pancreatic tumor microenvironment. Thus, loss of ARF may contribute to tumor development not only through disruption of MDM2–p53 regulation but also through alterations in the tumor–stroma interaction [99, 100].

p14ARF alone can trigger tumor cell death and stimulate immune-related pathways. However, its reliance on intact p53 signaling and its relatively modest efficacy as a single agent limit its therapeutic potential, supporting its application primarily within combination approaches [101]. The identification of p53-independent functions of ARF suggests that its therapeutic restoration may have broader effects than those attributable solely to p53 reactivation, although the extent to which these mechanisms contribute to the efficacy of p14ARF gene therapy remains to be established [99, 100].

To address these limitations, combinatorial strategies have been explored. In our own work, using melanoma xenograft models, combined p14ARF and IFN-β therapy enhanced immunogenic cell death, increased dendritic-cell activation, and improved survival compared with p14ARF alone [102, 103]. Likewise, co-expression of p14ARF and p53 in different models, such as non-small cell lung cancer and colorectal adenocarcinoma cell lines results in greater tumor suppression than either strategy alone [104], particularly when combined with agents such as aloperine, underscoring the benefit of coordinated pathway reactivation [105]. In another strategy, bicistronic adenoviral delivery of p14ARF with truncated BID under a tumor-responsive promoter enhanced apoptosis selectively in cells harboring oncogenic Ras and/or mutant p53, reinforcing the concept that contextual targeting improves specificity and efficacy [106].

Although p14ARF gene therapy is promising, important disadvantages and constraints remain. Therapeutic efficacy is highly dependent on intact downstream p53 signaling and cellular apoptotic competence. Tumor heterogeneity, oncogenic signaling context, and immune microenvironment factors can attenuate response. Furthermore, monotherapy approaches show limited capacity to induce sustained immune-mediated tumor control, underscoring the need for rational combinations.

Overall, current preclinical evidence supports p14ARF gene therapy primarily as a sensitizing and amplifying component within multimodal regimens. Its greatest potential appears to lie in integrated strategies that couple tumor suppressor restoration with immunomodulatory or pro-apoptotic mechanisms, rather than in standalone application.

### MDM2 as a potential target for therapy

MDM2 amplification and overexpression are recurrent alterations across multiple malignancies and are widely recognized as oncogenic events. Its canonical role involves direct binding to p53 and promotion of p53 ubiquitination and proteasomal degradation, thereby suppressing transcriptional programs governing cell-cycle arrest and apoptosis [107]. This *TP53*-dependent mechanism forms the basis for classical therapeutic strategies designed to disrupt the MDM2–p53 interaction using peptide-based or small-molecule antagonists that restore p53 activity [53]. However, these approaches depend on the presence of functional WTp53.

Beyond its regulation of p53, MDM2 exerts oncogenic effects independently of *TP53* status. It can promote degradation of Rb family proteins, facilitate E2F-driven S-phase progression, modulate E2F1 activity, and influence apoptotic signaling through interactions with factors such as FOXO transcription factors [53]. MDM2 also contributes to genomic instability through effects on DNA repair and checkpoint control and has been implicated in epithelial-mesenchymal transition and metastasis via regulation of SNAIL, TWIST, and TGF-β-associated pathways [108]. Clinically, MDM2 overexpression correlates with resistance to chemotherapy, radiotherapy, and certain targeted agents, including tyrosine kinase inhibitors, even in tumors lacking wild-type *TP53* [109, 110].

Additionally, MDM2 is a complex protein that undergoes extensive alternative splicing, generating multiple transcript variants with distinct biological functions. Several alternatively spliced isoforms, including MDM2-ALT1, MDM2-ALT2, and MDM2-ALT3, have been detected in a variety of human malignancies and have been associated with tumor progression, aggressive disease phenotypes, and poor clinical outcomes. Emerging evidence indicates that some of these truncated isoforms possess intrinsic oncogenic activity and may contribute to tumorigenesis independently of full-length MDM2 [111]. The presence of MDM2 splice variants may also represent a potential challenge for therapeutic strategies targeting the canonical MDM2–p53 interaction. Because several isoforms lack the N-terminal p53-binding domain, they may not be effectively targeted by small molecules designed to disrupt MDM2–p53 binding. Furthermore, some splice variants have been reported to exert p53-independent oncogenic functions, suggesting that inhibition of the canonical MDM2–p53 axis alone may not fully suppress MDM2-driven tumor-promoting activities [53, 111].

Given this multifaceted role, integrative therapeutic strategies extend beyond simple p53 reactivation. Three principal approaches have been explored. The first centers on inhibiting the MDM2–p53 interaction to stabilize p53 in *TP53* wild-type tumors [53]. The second aims to directly reduce MDM2 protein levels or restore upstream physiological regulators such as p14ARF, thereby attenuating MDM2 activity independent of small-molecule blockade. The third encompasses combination strategies that simultaneously target parallel survival and stress-response pathways influenced by MDM2, seeking to overcome compensatory signaling and therapeutic resistance. Currently there are no clinical protocols testing direct MDM2 targeting by gene therapy, but several pharmacological interventions targeting MDM2 are being tested (please see Table 1).

Inhibiting MDM2 with gene therapy may allow context-specific intervention in tumors characterized by MDM2 amplification, functional p53 suppression, or p53-independent oncogenic signaling. However, therapeutic impact remains biologically contingent on tumor genotype, pathway integrity, and adaptive resistance mechanisms. Collectively, available evidence supports targeting MDM2 not as an isolated intervention, but as part of rational, multi-target strategies designed to address both *TP53*-dependent and independent oncogenic functions [53, 107, 108].

### The potential for gene therapy targeting MDM2

Therapeutic targeting of MDM2 through genetic approaches has expanded beyond conventional small-molecule inhibition, incorporating gene restoration, gene silencing, and immunogenetic strategies. These approaches aim to counteract the oncogenic activity of MDM2, such as by the restoration of p14ARF, described above, reduce MDM2 expression or stimulate immune recognition of tumor cells over-expressing MDM2.

One integrative gene-silencing strategy combines plasmid-mediated delivery of siRNA directed against MDM2 with exogenous *TP53* gene transfer and zinc supplementation [112]. In this system, the siRNA component reduces endogenous MDM2 levels, thus limiting its impact on its molecular targets, including p53. Concurrently, introduction of a *TP53* expression cassette increases intracellular p53 availability. Zinc supplementation supports structural stabilization of the p53 DNA-binding domain, particularly in tumors harboring partially misfolded p53, facilitating recovery of transcriptional activity. In preclinical models, MDM2-directed siRNA combined with *TP53* gene transfer and zinc supplementation increased p53 stabilization and transcription of pro-apoptotic targets and enhanced apoptosis. In animal models, this combination was associated with inhibition of tumor growth [112].

Beyond combined MDM2 silencing and p53 restoration, multiple studies have explored siRNA- and shRNA-mediated MDM2 suppression as a therapeutic strategy. These approaches can reduce MDM2 expression and thereby alleviate its oncogenic effects, offering an alternative to pharmacological MDM2 inhibition. Depending on the cellular and molecular context, MDM2 depletion can interfere with several processes involved in tumor maintenance, including cell-cycle progression, survival, proliferation, and resistance to apoptosis. Importantly, emerging evidence indicates that the antitumor effects of MDM2 inactivation are not necessarily restricted to tumors with functional p53. In cell linen, MDM2 depletion reduces proliferation and survival, while in xenograft models, genetic suppression of MDM2 inhibits tumor growth, including in settings with compromised p53 activity, indicating that MDM2 can contribute to tumorigenesis through mechanisms that extend beyond its canonical negative regulation of p53. These findings suggest that MDM2 silencing may affect additional oncogenic pathways and cellular processes that remain relevant in p53-deficient tumors, although the specific mechanisms may vary according to tumor type and genetic background. Thus, MDM2-directed gene silencing may represent a broader therapeutic strategy than approaches designed solely to reactivate p53, potentially extending its applicability to tumors harboring mutant, inactive, or otherwise compromised p53. This can be particularly relevant for heterogeneous malignancies in which MDM2 amplification or overexpression may coexist with different *TP53* alterations [113–115].

From a strategic perspective, this combinatorial design addresses multiple nodes of the same pathway: suppression of a negative regulator (MDM2), restoration of tumor suppressor function (*TP53*), and structural support for protein conformation (zinc). The main advantages include mechanistic synergy and potential applicability in tumors with dysfunctional but not fully inactivated p53. However, limitations include dependence on efficient gene delivery, potential variability in zinc responsiveness, and the requirement for at least partially functional p53 signaling to achieve maximal therapeutic benefit.

Beyond intracellular modulation, genetic immunotherapy has introduced vaccine-based strategies targeting tumor-associated antigens, including MDM2. The plasmid DNA vaccine STEMVAC was developed to elicit immune responses against multiple antigens frequently overexpressed in solid tumors: MDM2, CD105, SOX2, YB-1, and CDH3. These targets are implicated in proliferation, stemness, angiogenesis, invasion, and therapeutic resistance. By incorporating multiple antigens, this platform seeks to broaden immune recognition and reduce immune escape driven by tumor heterogeneity. Early clinical studies have demonstrated induction of antigen-specific, Th1-skewed T-cell responses with an acceptable safety profile. Ongoing phase II trials are evaluating immunologic and clinical outcomes in breast cancer and non–small cell lung cancer (NCT05242965; NCT05455658; NCT07112053; NCT07078604).

Compared with direct gene replacement or silencing, vaccine strategies offer systemic immune-mediated tumor control and potential durability through immunological memory. Advantages include multi-antigen targeting and reduced reliance on tumor-intrinsic *TP53* status. Disadvantages involve variability in host immune competence, delayed onset of effect compared with cytotoxic gene strategies, and potential immune tolerance mechanisms.

Collectively, gene-based targeting of MDM2 encompasses intracellular modulation (silencing and restoration approaches) and immune-directed vaccination. Integration prospects include combining gene silencing or *TP53* restoration with immunotherapeutic platforms to simultaneously reduce oncogenic signaling and enhance tumor immunogenicity. Such combinatorial frameworks aim to overcome compensatory survival pathways and tumor heterogeneity while maintaining alignment with mechanistic evidence reported in preclinical and clinical investigations [112].

Overall, the evidence supporting gene-based targeting of the MDM2/p53/p14ARF axis spans multiple experimental levels. Studies in cancer cell lines primarily provide mechanistic evidence for transgene expression, pathway reactivation, apoptosis, and growth inhibition, whereas xenograft and other animal models provide evidence of tumor growth control, survival benefit, and modulation of the tumor microenvironment. In contrast, clinical gene therapy evidence remains concentrated on p53-directed approaches, particularly adenoviral p53 gene therapy and selected non-viral platforms, while clinical evidence for genetic MDM2 inhibition or p14ARF restoration remains limited. This distinction is important when considering the translational maturity of each strategy.

## Translational potential and clinical challenges

### Clinical application of integrated gene therapy: challenges and safety concerns

Targeted gene therapies directed at the MDM2/p53/p14ARF axis represent a rational strategy for cancer treatment. Although laboratory studies of genetic interventions that inhibit MDM2 or restore p14ARF are encouraging, clinical translation remains limited by biological, technical, and regulatory barriers. Many of these challenges overlap with those faced by other gene therapy approaches, yet some are intrinsic to modulation of this regulatory network.

A principal obstacle is the efficient delivery of therapeutic components to tumor tissue. Most strategies approaching clinical application depend on viral vector-mediated gene transfer [116–118]. Despite advances in vector engineering and improved targeting specificity, penetration into the often dense and heterogeneous tumor microenvironment remains suboptimal, limiting homogeneous intratumor distribution [119–121]. To address this issue, some experimental strategies aim to stimulate the patient antitumor immunity, thereby reducing the requirement for uniform gene delivery. Approaches combining expression of p53-related genes can enhance immunogenic cell death and antigen release [122–125], and combinatorial regimens with immune checkpoint inhibitors are under investigation [126].

Alternatively, pharmacological restoration of the p53 pathway using small molecules may offer improved tissue distribution compared with gene delivery systems [127–129]. However, systemic exposure raises concerns regarding off-target effects and toxicity in normal tissues, particularly given the central role of p53 in non-malignant cell homeostasis [130]. Strategies such as intratumor administration, tumor-responsive promoters, and improved vector specificity are therefore being explored to mitigate systemic risks.

For viral vectors targeting MDM2 or encoding p14ARF, immunogenicity and off-target transduction remain significant limitations [69, 131]. Adenoviral platforms, frequently used in clinical studies, often require intratumor administration due to hepatotoxicity and systemic inflammatory responses, restricting their use in systemic delivery approaches. Pre-existing immunity to adenoviruses, resulting from natural infections or vaccination, may further reduce therapeutic efficacy and safety [132–134]. Nonetheless, substantial progress in vector modification has led to the development of next-generation, less-immunogenic platforms, including adenovirus serotype 5 variations highly modified to avoid those issues [133–136].

Refinement of expression cassettes constitutes an additional strategy to enhance safety and mitigate unwanted effects. Certain regulatory elements intrinsic to the p53/MDM2 axis could be incorporated into expression cassette design or serve as inspiration for the development of synthetic regulatory elements. The p53–MDM2 axis itself provides a naturally occurring feedback mechanism, in which p53 induces MDM2 expression, while MDM2 negatively regulates p53 activity. In contrast, the PG promoter is a synthetic p53-responsive regulatory element that can be exploited to establish feedback-based expression systems. When used to drive p14ARF, PG can establish a synthetic positive-feedback mechanism, as p14ARF inhibits MDM2 and thereby enhances p53 activity, further stimulating PG-dependent expression. Similarly, when PG is used to drive p53 itself, an autoregulatory positive-feedback loop is established, in which p53 activates its own expression [137–141]. The study of such autoregulatory elements could lead to the development of context-dependent gene expression cassettes. Other inducible promoter systems are responsive to small molecules, light, or microenvironmental cues such as hypoxia, may provide mechanisms to modulate transgene activity and reduce unintended effects [142–146]. Although not all of them have been applied specifically to therapies targeting the MDM2/p53/p14ARF axis, they offer adaptable platforms for controlled gene modulation.

Another important challenge arises from *TP53* mutations. Genetic alterations in the DNA-binding domain not only abolish the canonical tumor-suppressive functions of wild-type p53 complex but may also cause toxicity through gain-of-function, a process also known as neofunctionalization [147, 148]. This process occurs when mutant p53 acquires novel pro-oncogenic activities beyond the mere loss of tumor suppression. A partially functional or mutant p53 can even promote the transcription of proto-oncogenes, rather than simply losing its wild-type properties, driving proliferation, genomic instability, metabolic rewiring, inflammation, invasion, and therapy resistance. These effects often result from structural changes that stabilize the mutant protein and enable aberrant interactions with DNA and other transcriptional regulators, thereby activating oncogenic transcriptional programs and contributing to tumor progression and cellular toxicity [149, 150].

Collectively, while therapeutic targeting of the MDM2/p53/p14ARF axis remains biologically compelling, successful clinical implementation will depend on overcoming delivery constraints, minimizing systemic toxicity, refining regulatory control of transgene expression, and accounting for the molecular heterogeneity imposed by *TP53* mutations.

### The importance of molecular stratification of patients

The MDM2/p53/p14ARF axis can be disrupted through diverse molecular mechanisms, including *TP53* point mutations [151, 152], *TP53* deletion, *MDM2* amplification or overexpression [153], CDKN2A/p14ARF loss, and alterations affecting other regulatory components of the pathway. Molecular stratification should therefore ideally consider the status of all three components. Interventions that aim to inhibit MDM2 or restore p14ARF each depend largely on *TP53* status and, as we argue here, may be best suited for tumor types that retain wild-type p53. Even so, *TP53* alterations represent the most extensively studied and clinically characterized biomarkers within this axis and provide a useful framework to illustrate how molecular heterogeneity can influence therapeutic responsiveness. Distinct genetic events, and even mutations affecting different structural domains of p53, can profoundly alter protein conformation, transcriptional activity, and treatment outcome. For instance, a mutation in a single *TP53* allele can compromise the remaining wild-type protein. Because p53 acts as a homotetramer, assuming comparable expression of both alleles, only one in sixteen tetramers would contain exclusively wild-type subunits, a dominant-negative effect that is allele- and target gene-dependent rather than absolute [154]. This requires co-oligomerization, however: mutations disrupting the tetramerization domain prevent mutant subunits from assembling with wild-type p53, allowing functional tetramers to still form.

Molecular stratification of the p53 axis, as well as the other associated genes, therefore, represents a critical step in optimizing therapeutic decision-making [155, 156]. Detailed characterization of *TP53* alterations, including defects in DNA binding, tetramerization capacity, or regulatory feedback, as well as assessment of genomic instability in associated pathway components, can guide rational selection of monotherapies or combinatorial regimens designed to overcome intrinsic resistance mechanisms [156]. This framework supports development of mutation-tailored strategies such as small molecules that restore wild-type-like p53 activity, allele-specific gene editing approaches, and immunotherapeutic modalities directed against mutant p53-expressing cells. Integration of comprehensive genomic profiling into clinical workflows enables identification of patient subgroups most likely to benefit from targeted interventions. This has the potential to enhance response rates, limit toxicity, and advance precision oncology [157]. Although *TP53* status is an extensively utilized stratification marker in current clinical and translational studies, other components of the MDM2/p53/p14ARF axis, as well as associated regulatory genes, are also relevant for the more precise selection of therapeutic strategies on a case-by-case basis.

### Integration with chemotherapy, radiotherapy, and immunotherapy

Activation of the MDM2/p53/p14ARF axis represents a clinically supported strategy to enhance the efficacy of chemotherapy, radiotherapy, and immunotherapy in *TP53* wild-type tumors by restoring DNA damage responses, lowering the apoptotic threshold, and promoting tumor-immune activation [19, 36, 41, 53, 158]. Reactivation of p53 increases tumor susceptibility to genotoxic stress, improving chemosensitivity and radiosensitivity while limiting tumor cell survival and repopulation.

We also must consider that the p53 pathway is essential for physiological stress responses in normal tissues, particularly within highly proliferative compartments such as the bone marrow and hematopoietic system. Therefore, therapeutic modulation of the MDM2/p53/p14ARF axis through existing platforms, such as MDM2 inhibitors and p53 gene therapy, may be associated with dose-limiting toxicities and off-target effects in non-malignant cells. This context reinforces the need for tumor-selective delivery systems and tightly regulated expression strategies, a necessity that many gene therapy platforms aim to address by improving targeting specificity and reducing unintended activation of p53-dependent apoptosis in healthy tissues.

In addition to its effects on cell-intrinsic tumor suppression, p53 activation enhances immunogenic cell death and promotes a more permissive tumor microenvironment, supporting improved responses to immune checkpoint blockade. This dual role, potentiating both cytotoxic therapies and antitumor immunity, positions p53 reactivation as a central integrator of multimodal treatment strategies [31, 36, 38].

Clinical evidence from early-phase trials evaluating p53 gene therapy, pharmacologic reactivation, and MDM2 inhibitors supports the feasibility and therapeutic benefit of these combinations, particularly in biomarker-selected tumors with functional p53 suppression. Although large-scale randomized trials remain limited, current data reinforce that p53 activation is most effective when integrated into rational, biomarker-driven combination regimens rather than used as a standalone approach [159, 160].

## Conclusion

Gene therapy targeting p53 has emerged as a biologically grounded strategy in cancer treatment, given its central role in blocking tumor progression. However, genetic interventions aimed at restoring p14ARF function, together with strategies that inhibit or modulate MDM2 activity, represent complementary mechanisms capable of reactivating tumor-suppressive pathways, particularly in tumors retaining functional *TP53*. Few examples exist of the clinical translation of gene therapies that inhibit MDM2 or restore p14ARF. Even so, we argue here that although direct clinical translation of gene therapies specifically designed to inhibit MDM2 or restore p14ARF remains limited, preclinical evidence from cancer cell-line and xenograft models provides a mechanistic and therapeutic foundation for further translational development. The efficacy of these strategies as monotherapies remains highly dependent on tumor molecular context, including *TP53* status and the integrity of downstream signaling networks.

In this setting, combinatorial approaches offer clear advantages. The integration of gene therapy with complementary modalities, such as RNA interference-based strategies, co-delivery systems targeting multiple nodes of the pathway, and genetic immunotherapies including multi-antigen DNA vaccines, broadens the scope of intervention by simultaneously targeting distinct and, in some cases, p53-independent mechanisms. This multi-layered targeting increases the likelihood of overcoming key limitations associated with single-agent therapies, including tumor heterogeneity, adaptive resistance, and immune evasion, while enhancing the robustness and durability of antitumor responses.

Although gene therapies directly targeting p53 are already well established, the field is progressively shifting toward more sophisticated and integrated strategies. These next-generation approaches emphasize the rational combination of gene-based and immunological interventions, with the goal of achieving more effective and context-adapted modulation of MDM2-driven oncogenic networks. Together, these advances point toward a future in which gene therapy is not applied as a standalone modality, but as part of coordinated, multi-targeted therapeutic frameworks designed to improve clinical outcomes.

## Data Availability

No datasets were generated or analysed during the current study.
